# Complex Coacervation of Soy Proteins, Isoflavones and Chitosan

**DOI:** 10.3390/molecules22061022

**Published:** 2017-06-20

**Authors:** Yu-Hsuan Hsiao, Sheng-Yang Hsia, Yin-Ching Chan, Jung-Feng Hsieh

**Affiliations:** 1 Ph.D. Program in Nutrition & Food Science, Fu Jen Catholic University, Taipei 24205, Taiwan; ariel.66@yahoo.com.tw; 2 Department of Food Science, Fu Jen Catholic University, Taipei 24205, Taiwan; evolutionallen@hotmail.com; 3 Department of Food and Nutrition, Providence University, Shalu District, Taichung 43301, Taiwan; ycchan0302@gmail.com

**Keywords:** β-conglycinin, glycinin, isoflavones, chitosan, coacevation

## Abstract

In this study, the chitosan-induced coacervation of soy protein-isoflavone complexes in soymilk was investigated. Most of the soymilk proteins, including β-conglycinin (7S), glycinin (11S), and isoflavones, were found to coacervate into the soymilk pellet fraction (SPF) following the addition of 0.5% chitosan. The total protein in the soymilk supernatant fraction (SSF) decreased from 18.1 ± 0.3 mg/mL to 1.6 ± 0.1 mg/mL, and the pH values decreased slightly, from 6.6 ± 0.0 to 6.0 ± 0.0. The results of SDS-PAGE revealed that the 7S α’, 7S α, 7S β, 11S A3, and 11S acidic subunits, as well as the 11S basic proteins in the SSF, decreased to 0.7 ± 0.5%, 0.2 ± 0.1%, 0.1 ± 0.0%, 0.2 ± 0.2%, 0.2 ± 0.2% and 0.3 ± 0.2%, respectively. We also found that isoflavones in the SSF, including daidzein, glycitein, and genistein, decreased to 9.6 ± 2.3%, 5.7 ± 0.9% and 5.9 ± 1.5%, respectively. HPLC analysis indicated that isoflavones mixed with soy proteins formed soy protein-isoflavone complexes and were precipitated into the SPF by 0.5% chitosan.

## 1. Introduction

Chitosan is a naturally non-toxic, modified, food-compatible polymer that is a cationic polysaccharide produced by the deacetylation of chitin [1]. The structure of chitosan comprises units of d-glucosamine and (1,4)-linked 2-amino-deoxy-β-d-glucan that form a linear polysaccharide [2]. This polysaccharide has been employed in the food industry to improve the safety and shelf-life of food products [3]. The protein-polysaccharide interactions play an essential structure-controlling role in foods [4]. Chen et al. reported that chitosan can be used as a coagulant in the preparation of cheese, as it induced destabilization and coagulation of casein micelles via chitosan-milk protein interactions [5]. No et al. suggested that chitosan could to be used as a coagulant to interact with soy proteins in soymilk to form insoluble complexes in the preparation of tofu [6].

Tofu is a gel-like soybean food, and its preparation generally includes the coagulation of soymilk followed by molding and pressing. Soymilk is a colloidal solution that contains 3.6% protein, 2.9% carbohydrate, 2.0% fat and 0.5% ash [7]. Among the soy proteins, 7S and 11S are the two major proteins in soymilk [8]. The 7S protein is a trimeric protein composed of three subunits (i.e., α, α’, and β subunits) with molecular weights of approximately 76, 70 and 53 kDa, respectively [9]. The 11S protein is hexameric and consists of acidic and basic polypeptides and consists of five subunits: A1aB1b, A2B1a, A1bB2, A3B4 and A5A4B3 [10]. These 7S and 11S proteins are the major materials involved in gelling and coagulation following the addition of coagulant during the production of tofu.

Tofu is an excellent source of isoflavones. Prabhakaran et al. reported that 1317.88 ± 6.77 μg isoflavones/g dry tofu was precipitated into tofu during the tofu-making process [11]. These isoflavones included daidzin, glycitin, genistin, daidzein, glycitein and genistein, and the contents of these isoflavones were found to be 1022.19 ± 13.2, 111.52 ± 7.8, 1049.32 ± 15.4, 37.14 ± 0.77, 37.14 ± 0.77 and 16.90 ± 0.53 μg/g, respectively. A previous study reported that isoflavones, including daidzin, glycitin, genistin, malonyldaidzin, malonylgenistin, daidzein, glycitein and genistein, bind with the 7S and 11S proteins to form 7S-isoflavone and 11S-isoflavone complexes in soymilk [12]. Moreover, the addition of polysaccharides to soymilk, such as propylene glycol alginate and chitosan, induced the coacervation of the soy protein-isoflavone complexes [13].

Soy protein-chitosan interactions have garnered considerable attention due to their influence on the structure of foods [14]. Huang et al. indicated that the complex was formed through the electrostatic interactions between the amine groups of chitosan (–NH_3_^+^) and the carboxyl groups of soy proteins (–COO^−^), with hydrogen bonding also being involved in the soy protein-chitosan interaction [15]. As mentioned above, chitosan has the potential to coacervate soy proteins. However, the effects of chitosan-induced coacervation of soy protein-isoflavone complexes have not been described. Therefore, we utilized SDS-PAGE and HPLC to analyze the effects of chitosan on the coacervation of the soy protein-isoflavone complexes. The objective of this study was to investigate the coacervation of soy protein-isoflavone complexes by chitosan in soymilk.

## 2. Results and Discussion

### 2.1. Effects of Chitosan on the Coacervation of Soy Proteins

**Figure 1:**
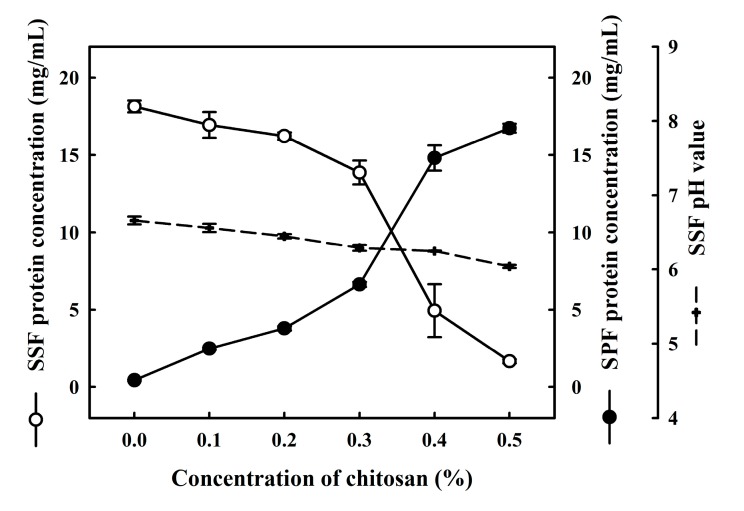
Changes in the total protein content of soymilk resulting following the addition of various quantities of chitosan (0.0%, 0.1%, 0.2%, 0.3%, 0.4%, or 0.5%). Samples were incubated at 30 °C for 1 h. ○: soymilk supernatant fraction (SSF); ●: soymilk pellet fraction (SPF). - - -: pH value (SSF). Vertical bars represent standard deviations.

Soymilk samples were incubated with various quantities of chitosan (0.0%, 0.1%, 0.2%, 0.3%, 0.4% or 0.5%) at 30 °C for 1 h, whereupon we determined the total protein content in both SSF and SPF. As shown in Figure 1, without the addition of chitosan, total protein levels in the SSF and SPF were 18.1 ± 0.3 and 0.4 ± 0.0 mg/mL, respectively. Previous studies have suggested that protein coacervation does not occur in soymilk samples without the addition of chitosan. However, we found that higher chitosan concentrations led to a decrease in protein content in the SSF and an increase in the protein content in the SPF. More specifically, 90.9 ± 0.4% of soy proteins were precipitated by chitosan, and the total protein concentration in the SSF decreased from 18.1 ± 0.3 mg/mL to 1.6 ± 0.1 mg/mL following treatment with 0.5% chitosan. Conversely, total protein in the SPF showed an obvious increase after the addition of 0.4% chitosan. In addition, the pH of each SSF sample treated with a different concentration of chitosan (0.0%, 0.1%, 0.2%, 0.3%, 0.4% or 0.5%) was 6.6 ± 0.0, 6.5 ± 0.0, 6.4 ± 0.0, 6.2 ± 0.0, 6.2 ± 0.0, and 6.0 ± 0.0, respectively. Our results revealed that most soy proteins coacervate at a pH of 6.0 and that the soy protein-chitosan ratio is approximately 3.6. Huang et al. previously reported that the maximum coacervation rate associated with the formation of the soy protein-chitosan complex occurred at a pH value of 6.0–6.5, 25 °C, and a ratio of soy proteins to chitosan of 4:1 was observed. The coacervation of soy proteins and chitosan is associated with interactions between proteins and polysaccharides [15]. Liu et al. reported that protein-polysaccharide interactions may be formed by an electrostatic attraction between the negatively charged surface of proteins and the positively charged chitosan [16]. Huang et al. further reported that these complex interactions involved hydrogen bonding between the amine groups of chitosan (–NH_3_^+^) and the carboxyl groups of soy proteins (COO^−^) [15]. Thus, the coacervation of soy proteins and chitosan is due to interactions between proteins and polysaccharides.

### 2.2. Analysis of the Effects of Chitosan on Soy Proteins Using SDS-PAGE

**Figure 2:**
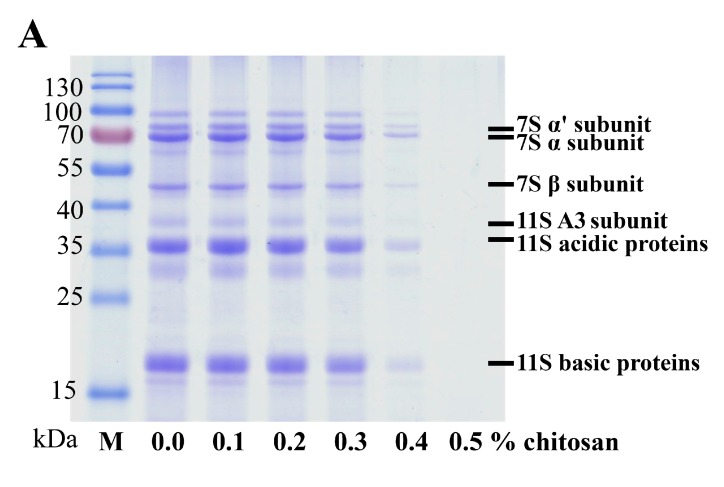

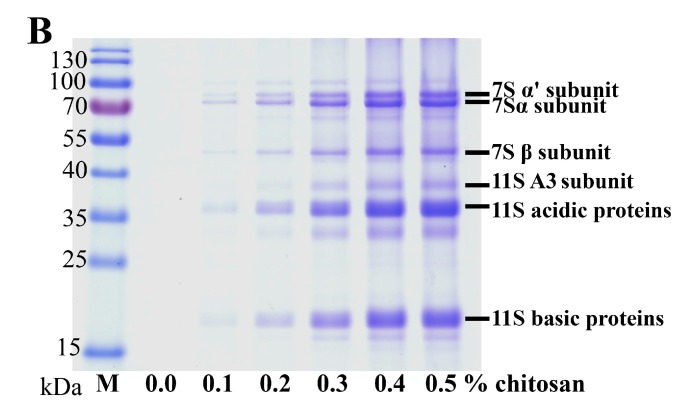
Changes in the SDS-PAGE profiles of soymilk following the addition of different amounts of chitosan (0.0%, 0.1%, 0.2%, 0.3%, 0.4%, or 0.5%). Samples were incubated at 30 °C for 1 h. (**A**) soymilk supernatant fraction (SSF); (**B**) soymilk pellet fraction (SPF); M: protein marker.

Glycinin globulin (11S) and β-conglycinin globulin (7S) are the two major proteins in soymilk [17]. Soymilk samples were incubated using various concentrations of chitosan (0.0%, 0.1%, 0.2%, 0.3%, 0.4% or 0.5%). The results of SDS-PAGE (Figure 2) indicate that the concentrations of the subunits (7S α’, 7S α, 7S β, 11S A3, 11S acidic, and 11S basic) in the SSF decreased as the concentration of chitosan increased (Figure 2A). Furthermore, a marked decrease in the concentration of proteins was observed at a chitosan concentration of 0.4%. Similarly, the quantities of soy protein subunits mentioned above increased in the SPF as the concentration of chitosan increased (Figure 2B). Therefore, our results suggest that a chitosan concentration of at least 0.4% could cause the agglomeration of soy protein subunits, including the 7S α’, 7S α, 7S β, 11S A3, 11S acidic, and 11S basic subunits, resulting in the transformation of SSF to SPF.

**Figure 3:**
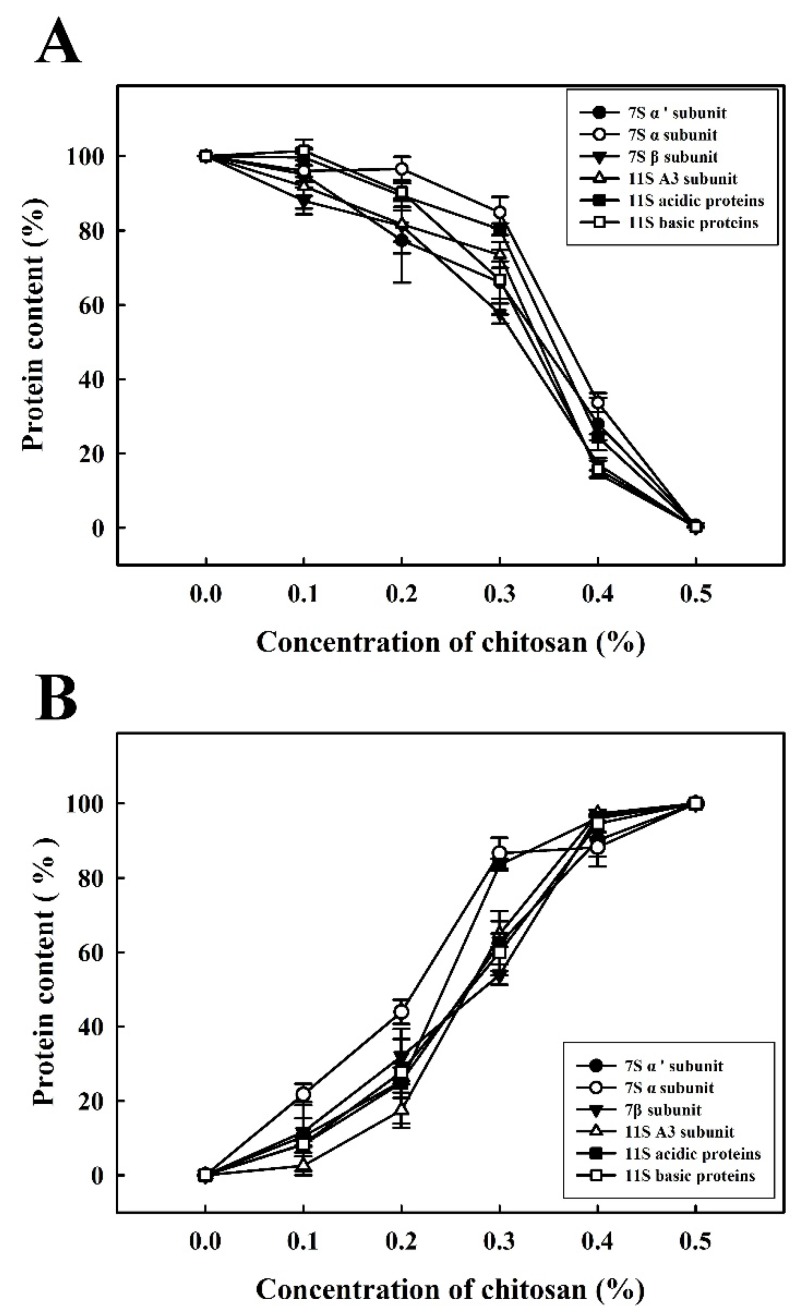
Densitograms corresponding to the SDS-PAGE analysis of soymilk proteins containing various concentrations of chitosan (0.0%, 0.1%, 0.2%, 0.3%, 0.4% or 0.5%). Samples were incubated at 30 °C for 1 h. (**A**) soymilk supernatant fraction (SSF); (**B**) soymilk pellet fraction (SPF).

Densitograms corresponding to the SDS-PAGE analysis of the soymilk samples treated with various quantities of chitosan are shown in Figure 3. When 0.4% chitosan was added, the intensities of total soy protein subunits sharply decreased. Moreover, at the highest concentration of chitosan tested (0.5%), the 7S α’, 7S α, 7S β, 11S A3, 11S acidic, and 11S basic subunits were decreased in the SSF to 0.7 ± 0.5%, 0.2 ± 0.1%, 0.1 ± 0.0%, 0.2 ± 0.2%, 0.2 ± 0.2% and 0.3 ± 0.2%, respectively (Figure 3A).

In addition, the intensities of the 7S α’, 7S α, 7S β, 11S A3, 11S acidic, and 11S basic subunits increased markedly in the SPF upon the addition of 0.4% chitosan (Figure 3B). Yuan et al. reported that chitosan is positively charged at a slightly acidic pH (<6.5) and that the carboxyl groups of proteins had a distributed negative charge that could be shown by the zeta-potential distribution [18]. The coacervation results also described the solubility of the 7S and 11S proteins following the addition of chitosan. Yuan et al. discussed the coacervate of the 7S protein and 11S protein combined with chitosan by turbidimetric analysis individually. They suggested that interactions between the chitosan-induced 7S proteins and the chitosan-induced 11S proteins become stronger at pH values of 6.0 and 6.5, respectively [19]. Nevertheless, the results of this study suggest that the 7S protein and 11S proteins coacervate by the addition of 0.5% chitosan and a decrease in pH to 6.0 ± 0.0.

### 2.3. HPLC Analysis of Isoflavones in Soymilk

**Figure 4:**
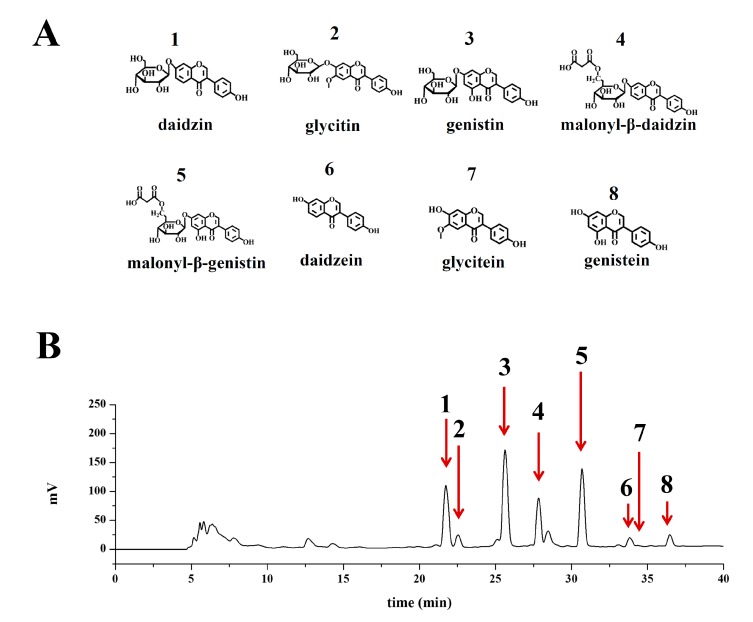
HPLC chromatograms: (**A**) isoflavone standards; (**B**) isoflavones extracted from soymilk.

Eight isoflavones (Figure 4A) were identified in soymilk by HPLC, and the HPLC chromatograph of soymilk is presented in Figure 4B. For this investigation, we separated the eight major isoflavones in soymilk samples into two types: glycones and aglycones. The glycone isoflavones include daidzin (peak 1), glycitin (peak 2), genistin (peak 3), malonyldaidzin (peak 4), and malonylgenistin (peak 5). The aglycones isoflavones were daidzein (peak 6), glycitein (peak 7), and genistein (peak 8) [12].

Wang et al. previously reported on the content of each isoflavone in 5.5 L soymilk; specifically, the daidzin, glycitin, genistin, malonyldaidzin, malonylgenistin, daidzein, glycitein, and genistein amounts were 65.6, 18.9, 90.2, 15.3, 48.5, 15.1, 7.9 and 19.7 mg, respectively [20].

### 2.4. HPLC Analysis of the Effects of Chitosan on the Isoflavones

**Figure 5:**
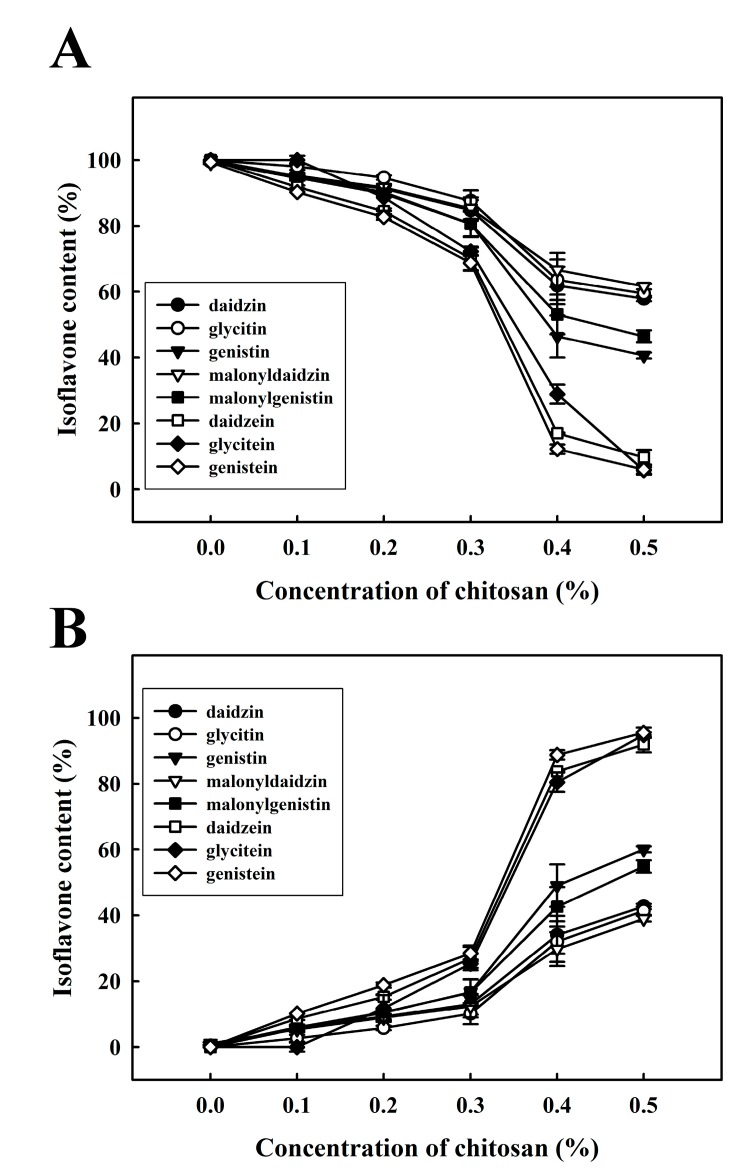
Changes in the percentage of isoflavone content (%) in soymilk samples with various quantities of chitosan (0.0%, 0.1%, 0.2%, 0.3%, 0.4% or 0.5%). Samples were incubated at 30 °C for 1 h. (**A**) soymilk supernatant fraction (SSF); (**B**) soymilk pellet fraction (SPF). Vertical bars represent standard deviations.

To understand the content of isoflavones transforming, we generated HPLC chromatographs to determine the isoflavone content in SPF treated with various concentrations of chitosan (0.0%, 0.1%, 0.2%, 0.3%, 0.4% or 0.5%), the results of which are shown in Figure 5. As previously mentioned, soy proteins in the SSF were found to coacervate into the SPF following the addition of 0.4% chitosan. Similar results were obtained when quantifying the isoflavone content that had transformed into SPF by the addition of 0.4% chitosan (Figure 5).

Conversely, when 0.4% chitosan was added to soymilk, the isoflavone content in the SSF began to decrease. When 0.5% chitosan was added to the SSF, the content of each isoflavone, including daidzin, glycitin, genistin, malonyldaidzin, malonylgenistin, daidzein, glycitein, and genistein, decreased to 21.0 ± 0.0, 2.7 ± 0.0, 12.4 ± 0.0, 21.1 ± 0.2, 25.3 ± 0.1, 0.2 ± 0.0, 0.0 ± 0.0 and 0.1 ± 0.0 μg/mL, respectively. These results reveal that the isoflavone content in the SSF decreases with the addition of chitosan. Conversely, the concentration of each isoflavone in the SPF increased with the concentration of chitosan. Specifically, the percentage of isoflaovnes in the SSF, including daidzin, glycitin, genistin, malonyldaidzin, malonylgenistin, daidzein, glycitein, and genistein decreased to 57.9 ± 0.8%, 59.4 ± 1.2%, 40.6 ± 0.9%, 61.6 ± 0.8%, 46.4 ± 1.8%, 9.6 ± 2.3%, 5.7 ± 0.9% and 5.9 ± 1.5%, respectively, when treated with 0.5% chitosan (Figure 5A). The content of glycone isoflavones (peaks 1–5) decreased by approximately 40–60%, and the content of aglycone isoflavones (peaks 6–8) decreased by approximately 90–95%. Therefore, the isoflavone content increased in the SPF with the addition of 0.4% chitosan and increased markedly with the addition of 0.5% chitosan (Figure 5B).

**Figure 6:**
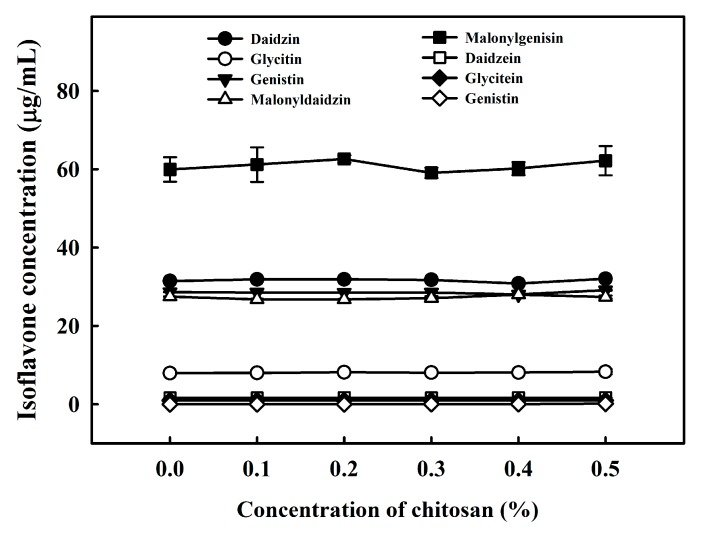
Changes in the isoflavone contents with different amounts of chitosan (0.0%, 0.1%, 0.2%, 0.3%, 0.4% or 0.5%) at 30 °C for 1 h. Vertical bars represent standard deviations.

The effects of chitosan on the coacervation of isoflavones were also evaluated. The isoflavones extracted from soymilk were incubated with 0.0–0.5% of chitosan. However, no significant changes in the amounts of daidzin, glycitin, genistin, malonyldaidzin, malonylgenistin, daidzein, glycitein, and genistein in the supernatant were observed (Figure 6). This result indicated that isoflavones did not immediately coacervate with chitosan. As previously mentioned, isoflavones, including daidzin, glycitin, genistin, malonyldaidzin, malonylgenistin, daidzein, glycitein, and genistein, were isolated from the dried 7S and 11S proteins [12]. These isoflavones bind to the 7S and 11S proteins to form 7S-isoflavone complexes and 11S-isoflavone complexes. These soy protein-isoflavone complexes then coacervate into the SPF following the addition of chitosan. The presence of soy protein-isoflavone complexes suggests that soy proteins and isoflavones are coprecipitated into tofu during the tofu-making process.

### 2.5. Reaction Scheme for the Effects of Chitosan on Soy Proteins and Isoflavones

**Figure 7:**
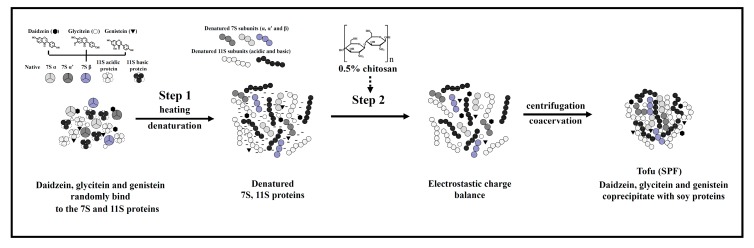
Reaction scheme illustrating the effects of chitosan on the coacervation of β-conglycinin, glycinin, and isoflavones in soymilk.

A reaction scheme based on the results of this study is presented in Figure 7. This scheme illustrates the effects of chitosan on the coacervation of individual soy proteins. The mechanism by which chitosan induced the coacervation of soy proteins involves two steps, as follows: (1) the hydrophobic regions of protein molecules in the native state are exposed to the outside environment by heat denaturation; (2) by adding chitosan, the chitosan is induced under a pH value of approximately 6.0 to be positively charged. The negative charge of the carboxyl groups of soy proteins forms a complex with the positively charged amine groups of chitosan through an electrostatic interaction [21]. When charge balance occurs, the soy proteins and chitosan molecules coacervate through hydrophobic interactions [19]. Furthermore, hydrogen bonding is also involved with coacervation [15]. Finally, the isoflavones induced by chitosan in soymilk coacervation are coprecipitated with proteins, and hydrophobic interactions cause most aglycones to coacervate with soy proteins, while only half of the glycones coacervate with the 7S and 11S proteins.

## 3. Experimental Section

### 3.1. Preparation of Soymilk

Soybeans [*Glycine max* (L.) Merrill, 100 g] were washed and soaked in distilled water at 25 °C for 12 h. The hydrated seeds were drained and ground into homogenates in 1 L of distilled water. The raw soymilk was filtered through a cotton filter and heated in a 90 °C water bath for 1 h. The soymilk was collected and stored at 4 °C. To investigate the effects of chitosan on the aggregation of soymilk proteins, soymilk was centrifuged at 12,000× *g* for 10 min at 4 °C using a centrifugal separator to remove the lipids. The defatted filtrate was used as soymilk in subsequent experiments.

### 3.2. Preparation of Soymilk Samples Containing Various Concentrations of Chitosan

Chitosan oligosaccharide lactate (molecular weight: 4–6 kDa, deacetylation > 90%) was purchased from Sigma Chemical Co. (St. Louis, MO, USA). To investigate the effects of chitosan on the coacervation of soymilk proteins, soymilk samples with varying amounts of chitosan (0.0%, 0.1%, 0.2%, 0.3%, 0.4% or 0.5%) were incubated at 30 °C for 1 h. After incubation, the soymilk samples were fractionated into the soymilk supernatant fraction (SSF) and the soymilk pellet fraction (SPF) by centrifugation for 20 min (12,000× *g*). The protein concentrations of the SSF and SPF samples were determined using a protein assay kit (Bio-Rad, Hercules, CA, USA). The Bio-Rad protein assay dye was diluted with 4 volumes of water; then, it was mixed with individual standards or soymilk samples. The absorbance at 595 nm was measured using a VersaMax™ microplate reader (Molecular Devices Corporation, Sunnyvale, CA, USA), and bovine serum albumin (Sigma Chemical Co.) was used as the standard.

### 3.3. Sodium Dodecyl Sulfate Polyacrylamide Gel Electrophoresis (SDS-PAGE)

Soymilk samples were analyzed using SDS-PAGE, in accordance with protocol outlined by Hsiao et al. [13]. Electrophoresis was carried out in 1.5 mm thick gels that contained a 5% stacking gel (*w*/*v*) and a 12.5% separating gel (*w*/*v*). For each soymilk sample, a 0.1-mL volume of sample was mixed with 0.3 mL of buffer (2% SDS, 5% β-mercaptoethanol, 10% glycerol, 0.02% bromophenol blue and 70 mM Tris-HCl, pH 6.8) and was heated to 95 °C for 5 min. The samples (4 µL) and a protein ladder were loaded into separate wells of the gels. Following electrophoresis, the gels were stained with Coomassie Brilliant Blue R-250. The stained gels were imaged using an EPSON perfection 1270 image scanner (Epson America Inc., Long Beach, CA, USA) and analyzed using the Gel-Pro Analyzer (version 4.0, Media Cybernetics, Inc., Rockville, MD, USA) software program.

### 3.4. Preparation of Isoflavone Samples

Soymilk, SSF, and SPF samples were lyophilized into powders to extract isoflavones in accordance with the method described by Prabhakaran et al. [11]. Specifically, 80% methanol was added to freeze-dried samples to serve as an extraction solvent, and the solution was shaken in a vortex mixer at 30 °C for 2 h. After a 10 min centrifugation at 12,000× *g* at 4 °C, the supernatants (isoflavones) were subjected to high-performance liquid chromatography (HPLC) analysis.

### 3.5. HPLC Analysis of the Isoflavone Samples

Analysis of isoflavone samples (20 µL) was performed using a method modified from Tipkanon et al. [22]. The HPLC system comprised a PU-980 pump, and a UV-970 detector (both from JASCO, Tokyo, Japan) and a C18 packed column (Mightysil RP-18 GP, 4.6 mm × 250 mm, 5 μm spherical, Kanto Chemicals, Tokyo, Japan). The mobile phase of methanol (A) and water (B) was used with a gradient elution of 25% A and 75% B that was increased to 35% A after 10 min, 50% A after 25 min, 100% A after 35 min, and 100% A after 40 min. The flow rate was 0.5 mL/min with the column maintained at a temperature of 30 °C. Detection was conducted at a wavelength of 260 nm. Eight commercial isoflavones were analyzed as the standards. Daidzin, glycitin, genistin, daidzein, glycitein and genistein were purchased from LC Laboratories (Woburn, MA, USA), while malonyldaidzin and malonylgenistin were purchased from Nacalai Tesque, Inc. (Kyoto, Japan).

### 3.6. Statistical Analysis

SPSS statistical software (version 10.0.7C, SPSS Inc., Chicago, IL, USA) was used for statistical analysis. Data were expressed as the means ± standard deviations. The statistically significant differences between treatments were determined using one-way ANOVA followed by Duncan’s multiple range test. Three determinations for each treatment were performed, and the significance level was set at *p* < 0.05.

## 4. Conclusions

This study examined the effects of chitosan on the coacervation of β-conglycinin, glycinin and isoflavones in soymilk. The results of SDS-PAGE clearly demonstrate that most of the 7S (α’, α, and β), 11S acidic (A3) and 11S basic protein subunits in soymilk coacervate by the addition of 0.5% chitosan. Moreover, HPLC analysis indicates that 90–95% of the aglycones (including daidzein, glycitein and genistein) had interacted with the 7S and 11S proteins. These isoflavones were also coprecipitated into SPF by 0.5% chitosan.
